# The Evolution of the Specialist Surgeon Workforce in East, Central and Southern Africa

**DOI:** 10.1002/wjs.12545

**Published:** 2025-03-20

**Authors:** Denise Osei‐Kuffour, Chihena Hansini Banda, Alice Campion, Luli Baissa Gerba, Precious Mutambanengwe, Mugisha Nkoronko, Michael Mwachiro, Noel Aruparayil, Alliance Niyukuri, Godfrey Sama Philipo, Deirdre Nally, Deirdre Mangaoang, Eric Borgstein, Abebe Bekele

**Affiliations:** ^1^ Chelsea and Westminster Hospital London UK; ^2^ Plastic and Reconstructive Surgery Unit Department of Surgery The University Teaching Hospital Lusaka Zambia; ^3^ Great Western Hospital Swindon UK; ^4^ Ras Desta Damtew Memorial Hospital Ministry of Health of Ethiopia Addis Ababa Ethiopia; ^5^ Sally Mugabe Central Hospital Harare Zimbabwe; ^6^ Kilimanjaro Christian Medical Center Moshi Tanzania; ^7^ Avenue HealthCare Nairobi Kenya; ^8^ College of Surgeons of East Central and Southern Africa (COSECSA) Arusha Tanzania; ^9^ University of Leeds Leeds UK; ^10^ Mercy James Center for Paediatric Surgery and Intensive care Blantyre Malawi; ^11^ Research Department Mercy Surgeons Bujumbura Burundi; ^12^ Royal College of Surgeons in Ireland (RCSI) Dublin Ireland; ^13^ Institute of Global Surgery RCSI University of Medicine and Health Sciences Dublin Ireland; ^14^ University of Global Health Equity Kigali Rwanda

**Keywords:** global health, health workforce/trends, surgeons/supply & distribution, workforce

## Abstract

**Background:**

Access to surgery across sub‐Saharan Africa faces persistent challenges with substantial disparity between disease burden and the surgical workforce. This updated situational analysis of specialist surgeons was undertaken to monitor progress toward global surgery development goals and address workforce deficits.

**Methods:**

A cross‐sectional analysis of the surgeon workforce across 12 of the 14 member countries of The College of Surgeons of East, Central and Southern Africa (COSECSA) was conducted between 2021 and 2022. The data was validated by at least two sources, including medical council registers and direct contact with surgeons via COSECSA Country representatives. Results were compared to data collection undertaken in 2015.

**Results:**

2555 surgeons were identified as practising within the region, a 42% increase since 2015. This represents a rise of only 0.06 surgeons per 100,000. Surgeon density varies widely, with an 18‐fold difference between the lowest (Mozambique, 0.22/100,000) and the highest surgeon densities (Namibia, 3.97/100,000). Women surgeons constitute one‐tenth of the surgical workforce, a figure stagnant since 2015. Most surgeons (58%) practice in highly populated areas, and 78% work in their country of primary qualification.

**Conclusion:**

Currently there is a higher rate of population growth relative to surgical workforce expansion. Innovative approaches in surgical training are crucial to meet 2030 workforce targets. The non‐progression in the ratio of female to male surgeons demands attention. Future workforce planning should recognize the growing impact of female doctors on the healthcare workforce and prioritize strategies to support women in surgical careers.

## Graphical abstract

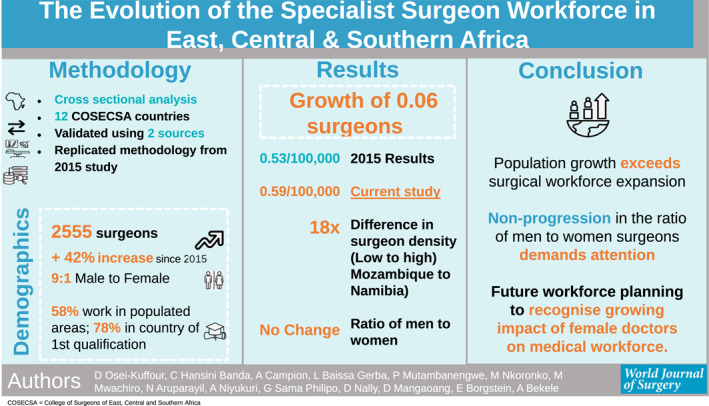

## 1 Introduction

Access to equitable surgical care is a significant challenge globally, particularly in Low‐ and Middle‐Income Countries (LMICs), where the poorest one‐third access only 6% of all surgeries performed worldwide [1, 2]. Although access to surgical care is multifactorial, the lack of adequate personnel with surgical expertise is a critical component.

Previous studies have acknowledged the high level of disparity between disease burden and health worker density. Africa has a quarter of the disease burden but only 3% of the world's health workforce [3]. The Lancet Commission on Global Surgery highlighted the vast variations which exist between and within countries [4] with greatest disparity in rural settings.

The College of Surgeons of East, Central and Southern Africa (COSECSA) was established in 1999 and is the largest surgical training body in the region [5]. The first analysis of the Specialist Surgeon Workforce was published in 2016, which found 1690 practising surgeons within 10 COSECSA‐member countries, creating an overall ratio of 0.53 surgeons per 100,000 [6]. Since then, the number of member countries has increased to 14. Different stakeholders have worked to improve the provision of surgery and surgical training, and several initiatives to retain surgeons have been implemented [7, 8, 9]. This study describes and analyses the change in the specialist surgical workforce from 2015 to 2022.

## 2 Methods

A tripartite collaborative research partnership was established between a trainee‐led global surgery interest group (Global Anesthesia, Surgery and Obstetric Collaboration, GASOC), the Royal College of Surgeons in Ireland (RCSI) Institute for Global Surgery, and COSECSA.

## 3 Study Population

### 3.1 Inclusion and Exclusion Criteria

All surgeons (COSECSA and non‐COSECSA trained) currently practising in the 14 COSECSA‐member countries were included, representing the following specialities: General surgery, Orthopedic surgery, Pediatric Surgery, Urology, Plastic Surgery, Ear Nose and Throat (ENT), Neurosurgery, Cardiothoracic Surgery and Oral and Maxillofacial surgery (OMFS). Surgeons who were retired, working within the subspecialties of ophthalmology and obstetrics and gynecology, or working part‐time or in a rotational capacity were excluded.

Ethical approval for this study was jointly obtained from the Institutional Review Boards of COSECSA and RCSI. COSECSA IRB ID: 00011122. RCSI IRB ID: 212573743. In addition, GDPR principles and data management strategies were adhered to.

## 4 Data Collection and Analysis

Data was collected based on the published methodology of O'Flynn et al. [6] and our results analyzed in comparison using descriptive statistics. Data verified from a minimum of two independent sources were included in the analysis. For a full list of sources, please see Appendix 1. Several sources were used to confirm the population size of countries, cities and towns [10, 11, 12]. South Sudan and Sudan were excluded from the analysis; Due to ongoing conflict, it was difficult to obtain accurate information on the names and locations of practising surgeons. Namibia and Botswana were excluded from figures comparing results between the two studies as these countries were not part of COSECSA in 2015. Data collection was completed in November 2022. Study data was collected and managed using REDCap electronic data capture [13, 14].

## 5 Results

### 5.1 General Overview

Overall, 2555 surgeons were identified as working within the COSECSA region (12 countries; Sudan and South Sudan excluded). In the 10 countries for which data was available in 2015, there has been an increase of 42% (Table 1) [6]. The most significant increase in surgical workforce numbers were seen in Ethiopia (89%) and Burundi (174%), which saw a rise of 2–3 times pre‐existing figures, respectively. Uganda was the only country demonstrating a reduction in the number of surgeons (13%). The ratio of men to women remained static at 9:1 (2298 men to 243 women). For further information on data completeness, see Appendix 2.

**TABLE 1:** The total number of surgeons identified as working within the COSECSA region listed by country. Data presented alongside 2015 data for comparison with the percentage change (—: No available data).

Country	Total number of surgeons		Change (%)
	2015	2022	
Botswana	—	57	—
Burundi	19	52	174
Ethiopia	337	636	89
Kenya	543	810	49
Malawi	41	53	29
Mozambique	57	72	26
Namibia	—	102	—
Rwanda	49	86	76
Tanzania	177	183	3
Uganda	259	225	−13
Zambia	85	116	36
Zimbabwe	123	163	33
Sudan	—	—	—
South Sudan	—	—	—
Total	1690	2555	42

*Note:* —: No available data.

### 5.2 Speciality Analysis

Speciality data was recorded for 95% of surgeons (Appendix 2) and is displayed in Table 2. General surgery remained the most prevalent surgical speciality (47%), followed by orthopedic surgery (20%) (Table 2). There was an 80% increase in the number of neurosurgeons from 69 to 124; The largest increase in any speciality. In addition, the number of ENT surgeons increased by 65%. On a country specific level, strikingly, there were 40 ENT surgeons in Ethiopia when none were recorded in 2015 [6]. Malawi and Zambia had the highest proportion of pediatric surgeons; 11% and 9% respectively. However, despite these individual speciality increases, the overall proportion of different specialities within the region remained unchanged.

**TABLE 2:** The total number of surgeons within each subspecialty by Country.

Distribution	General surgery	%	Orthopedic surgery	%	Urology	%	Plastic surgery	%	ENT	%	Neurosurgery	%	Pediatric surgery	%	OMFS	%	Cardiothoracics	%	Total surgeon speciality known	%	Not recorded	%	Total surgeons
Botswana	22	39	15	26	5	9	3	5	4	7	2	4	1	2	2	4	3	5	57	100	0	0	57
Burundi	30	58	9	17	5	10	0	0	4	8	2	4	1	2	0	0	1	2	52	100	0	0	52
Ethiopia	335	57	106	18	26	4	20	3	40	7	21	4	17	3	6	1	16	3	587	92	49	8	636
Kenya	355	46	163	21	41	5	16	2	93	12	42	5	17	2	37	5	15	2	779	96	31	4	810
Malawi	22	42	13	25	2	4	3	6	1	2	4	8	6	11	1	2	1	2	53	200	0	0	53
Mozambique	25	36	12	17	7	10	3	4	6	9	5	7	3	4	1	1	7	10	69	96	3	4	72
Namibia	36	35	22	22	11	11	3	3	8	8	7	7	1	1	9	9	5	5	102	400	0	0	102
Rwanda	40	47	16	19	7	8	2	2	14	16	5	6	2	2	0	0	0	0	86	100	0	0	86
Tanzania	56	38	45	31	14	10	3	2	6	4	9	6	4	3	0	0	10	7	147	80	36	20	183
Uganda	117	53	35	16	9	4	9	4	23	10	13	6	5	2	4	2	6	3	221	300	4	2	225
Zambia	58	52	24	22	9	8	2	2	3	3	4	4	10	9	0	0	1	1	111	96	5	4	116
Zimbabwe	49	30	34	21	16	10	5	3	19	12	19	12	8	5	9	6	4	2	163	100	0	0	163
TOTAL	1145	47	494	20	152	6	69	3	221	9	133	5	75	3	69	3	69	3	2427	95	128	5	2555

*Note:* Sudan = 0, South Sudan = 0.

### 5.3 Sex Analysis

**FIGURE 1:**
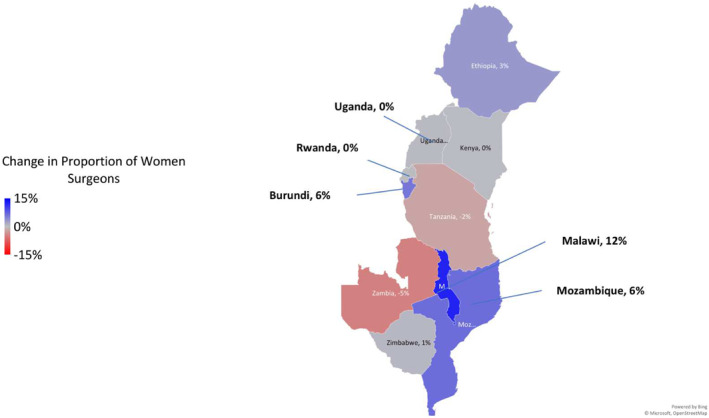
Change in proportion of Women Surgeons 2015 versus 2022.

Women surgeons comprised one‐tenth of the surgical workforce. The ratio of men to women surgeons was 9:1, unchanged since 2015 (Table 3; Supplemental Figure 1a). Malawi had the highest increase in women surgeons (12%) (Figure 1). Malawi (25%) and Mozambique (22%) had the largest proportion of women surgeons within their workforce (Supplemental Figure 1d). Speciality choice significantly varies amongst men and women surgeons (*p* < 0.0001) (Supplemental Table 1). The surgical specialities with the highest proportion of women were pediatric (28%) and plastic surgery (28%). Women were least represented in cardiothoracic surgery (3%), followed by orthopedics (5%) and urology (5%). By total numbers within the speciality, general surgery housed the most women surgeons (99 women).

**TABLE 3:** A table demonstrating the total number of surgeons in each Country categorized by sex and with comparison between data collected in 2015 and 2022.

Country	Male (2022)	Female (2022)	Total (2022)	% female	Male (2015)	Female (2015)	% Change in proportion of women surgeons	% Change in the actual number of women surgeons
Botswana	55	2	57	4%				
Burundi	49	3	52	6%	19	0	6%	—
Ethiopia	588	48	636	8%	322	15	3%	220%
Kenya	729	68	797	9%	499	44	0%	55%
Malawi	40	13	53	25%	36	5	12%	160%
Mozambique	56	16	72	22%	48	9	6%	78%
Namibia	94	8	102	8%				
Rwanda	77	9	86	10%	44	5	0%	80%
Tanzania	164	19	183	10%	156	21	−2%	−10%
Uganda	197	28	225	12%	228	31	0%	−10%
Zambia	99	16	115	14%	69	16	−5%	0%
Zimbabwe	150	13	163	8%	114	9	1%	44%
**Total**	**2298**	**243**	**2541**	**11%**	**1535**	**155**	**2%**	**50%**

*Note:* Bold denotes final totals.

There was significant correlation between sex and affiliation to COSECSA (Supplemental Figure 1b); more female than male surgeons obtained their primary surgical qualification through COSECSA (*p* < 0.0001). Overall, a higher proportion of women surgeons (43%) was affiliated with COSECSA than that of their male counterparts (32%). There was no association between hospital type (public or private sector working) and sex (Supplemental Figure 1c).

### 5.4 Surgical Workforce Density

There has been progress in the ratio of surgeons to population served. For the 12 countries included in both studies, there were 0.62 surgeons/100,000 of the population (Table 4). When considering the original 10 countries in the 2015 study, the total number increased by 42% from 1690 to 2396. However, when measured by surgeon: population ratio, the increase was more modest, rising from 0.53 to 0.59 per 100,000.

**TABLE 4:** Surgeon: Population Density with data from both 2022 and 2015 [12 Countries].

Countries	2022 population [15]	Total surgeons 2022	Ratio (per head of population)	Surgeons per 100,000 (2022)	Surgeons per 100,000 (2015)	% change
Botswana	2,630,296	57	46,146	2.17		
Burundi	12,889,577	52	247,876	0.40	0.18	55%
Ethiopia	123,379,925	636	193,994	0.52	0.35	32%
Kenya	54,027,487	810	66,701	1.50	1.21	19%
Malawi	20,405,317	53	385,006	0.26	0.24	8%
Mozambique	32,969,519	72	457,910	0.22	0.23	−5%
Namibia	2,567,013	102	25,167	3.97		
Rwanda	13,776,698	86	160,194	0.62	0.4	36%
Tanzania	65,497,748	183	357,911	0.28	0.36	−29%
Uganda	47,249,585	225	209,998	0.48	0.72	−51%
Zambia	20,017,675	116	172,566	0.58	0.58	0%
Zimbabwe	16,320,537	163	100,126	1.00	0.89	11%
TOTAL	411,731,377	2555				
Regional surgeon: Population ratio				161,147		
Regional surgeons per 100,000 population				0.62		

*Note:* Sudan and South Sudan excluded.

In Namibia, there were nearly 4 surgeons/100,000 and in Botswana there were 2 surgeons/100,000 (Table 4). These two countries joined COSECSA since the original study. However, in selected countries (Uganda, Tanzania, Mozambique, and Zambia), the ratio of surgeons/100,000 population had decreased. There is an 18‐fold difference between the country with the lowest ratio (Mozambique, 0.22/100,000) and that with the highest (Namibia, 3.97/100,000).

### 5.5 Country of First Surgical Qualification

**FIGURE 2:**
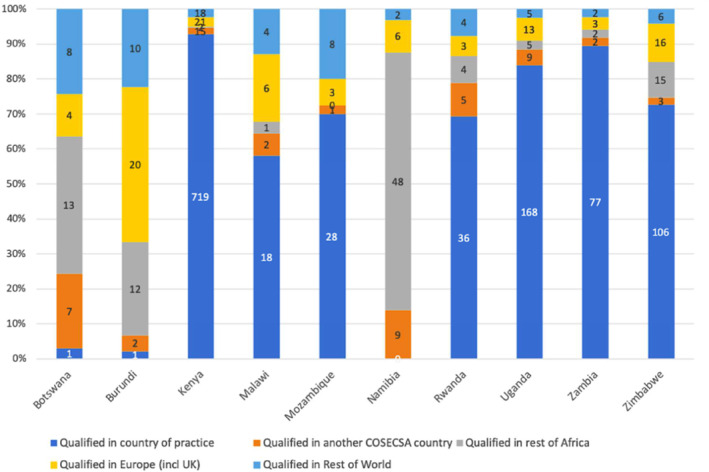
Surgeon Country of practice and location of First Surgical Qualification. Data collected for 58% of surgeons. (Ethiopia and Tanzania are not included in the table as a high percentage of surgeons practising in these countries did not have their ‘Country of first surgical qualification obtained’ recorded).

Information on the country of first surgical qualification was recorded for 1482 surgeons (58%). Of these, the majority qualified in their country of practice (*n* = 1162; 78%) (Figure 2). A further 56 (4%) qualified in another COSECSA‐member country, 102 in the rest of Africa (7%); 95 qualified in Europe (6%) and 67 in the rest of the World (5%).

### 5.6 Surgeon Location

Data regarding the location of employment was available for 92% of surgeons (Supplemental Table 2). Fifty‐eight percent of Surgeons were concentrated in areas with a population greater than 500,000. Botswana and Burundi had a higher proportion of surgeons working in smaller towns and cities. Due to a lack of data availability, surgeon location data was not analyzed for Uganda.

## 6 Discussion

Between 2015 and 2022, there was a marked increase (42%) in the number of practising surgeons but this was insufficient for the level of population growth that occurred within the same period. According to the United Nations [15], the total population of these 10 countries increased by 27%. Effectively, more than two‐thirds of the rise in the number of surgeons was necessary to keep pace with population growth. Therefore, the surgeon‐population ratio appears modest at 0.59 per 100,000, which is an increase of 0.06 surgeons per 100,000 since 2015 [6].

Several stakeholders have contributed to the rise in the number of specialist surgeons. The addition of the COSECSA collegiate training model to the pre‐existing university surgical training model has enabled a significant expansion in the number of newly trained surgeons [16, 17]. The support of international organizations such as the Global Surgery Foundation, the WHO and local non‐governmental organizations has encouraged the wider implementation of National, Surgical, Obstetric and Anesthesia Plans (NSOAPs) to help countries track progress toward workforce targets. The majority of plans were created after 2015 and they are in place or in process in most of the countries included in this study [18].

In Burundi, the threefold increase in surgeons could represent an underreporting of surgeons in the 2015 study; Burundi had recently joined COSECSA in 2012 [6]. We also observed a decrease in the number of surgeons in Uganda between 2015 and 2022, from 259 to 225. Whilst this could be attributable to difficulties faced accessing sources to validate surgeons in Uganda, it could also highlight that Uganda has been disproportionately affected by issues with health worker retention [19, 20, 21]. The Specialist Surgical Workforce in Uganda was validated using three key sources: the COSECSA “capsule” database [6], the Association of Surgeons in Uganda, and the Uganda Medical and Dental Practitioners Council register and attempts were made to contact every regional referral hospital in the country. The number of surgeons validated in Tanzania were lower than figures published in a recent review of NSOAP implementation in Tanzania [22]. Hellar et al. reproduced figures from the Medical Council of Tanganyika (MCT) database during the same period. Although there is a difference in methodology between the two studies (i.e. retired, part‐time or rotational qualified surgeons were excluded and pediatric surgeons were included), their published figures report 654 surgeons to our 183. Unfortunately, we were unable to engage with the MCT despite attempts to contact the society and the regional country representatives and therefore the figures presented here are the figures we were able to validate using the data sources available at the time.

Continued innovative training approaches are required to meet the target of 20 SAO personnel by 2030. As per the Lancet Commission in Global Surgery [4], the data in this study demonstrates regional disparity in surgeon‐to‐population density between East and Central African regions and Southern Africa. Therefore, tailored country specific strategies are needed. In Ethiopia, part public sponsorship of residency programmes and the expansion in their number has promoted significant strides in addressing workforce shortages [23].

KidsOR have helped expand the number of pediatric surgeons particularly in Malawi and Zambia by developing theaters and scholarships. Their target is to fund 120 pediatric surgeons by 2030 in partnership with WACS and COSECSA, with Southern Africa prioritized [24]. Despite the increase compared to the 2015 dataset, the overall ratio of men to women surgeons has remained consistently low at 9:1 [6]. This figure varies throughout the region, with a decrease in some countries. The men to women variation in relation to specialty choice is of interest. Historically, specialist surgical training in the region was focused on general surgery and later orthopedics. However, over the last decade, training in subspecialties such as neurosurgery and pediatric surgery has risen. The notably higher proportion of women surgeons in these newer subspecialties may reflect historical relics as well as the support of exclusive grants and scholarships [25, 26]. There has been considerable research into possible reasons for gender disparity in surgical career selection. Reasons cited include a lack of female role models, particularly in leadership positions, sociocultural norms, and an unfavorable training environment [27, 28, 29, 30]. A 2014 survey in Zimbabwe by Muchemwa et al. evaluated deterrent factors. Women cited concerns about the professional and physical demands of surgery. The study highlighted the trend between women with leadership traits and an interest in surgery. In 2022, COSECSA elected their first female president [31], and several female‐focussed initiatives and international collaborative ventures were introduced [25, 32, 33]. A key area for future intervention is the growing number of female medical students and their impact on the future surgical workforce. Displaying positive role models of women surgeons' success through widening participation (e.g. DREAM in Zimbabwe) and mentoring schemes is an achievable solution [34]. In 2015, Women in surgery Africa (WiSA) was established as an organization to encourage women surgeons and trainees [33].

However, in countries with a limited number of women surgeons, increasing mentorship and visibility of female leadership is less feasible. Therefore, it has been suggested that efforts might be better focused on challenging stereotypical views on gender roles and female education; This is an example of how tailored solutions are needed [35]. Altering socio cultural ideologies could take longer to influence, but it is a United Nations Sustainable Development Goal to “achieve gender equality and empower all women” [36].

Further qualitative and mixed methods research is needed to examine deterrents and successful measures [28, 29, 37, 38]. Overall, a collaborative approach is recommended to address the disparity in the sex ratio of surgeons. Regional and international organizations supporting surgery in Africa must recognize this disparity and actively work to address this through advocacy, innovative policy implementation and the support of regional programs such as WiSA, COSECSA as well as local women surgeon chapters.

### 6.1 Data Considerations and Limitations

This study has several limitations. Due to the civil unrest in Sudan and South Sudan, it was not possible to obtain accurate information on practising surgeons, and therefore they were discounted from the analysis. By only focusing on specialist surgeons, analysis of the contribution made by residents (surgeons in training), part‐time surgeons, non‐physician clinicians and non‐specialist physicians was restricted. The surgical workforce is continuously in flux, and it is difficult to accurately quantify the number of surgeons in any given country at any time. This study is a snapshot of the specialist surgeon workforce at a moment in time. Our study team has tried its best to accurately represent the current situation using available validated sources (see Appendix). However, the accuracy of this data is reduced over time. The figures we were able to validate may not be fully reflective of the active surgical workforce (particularly in Uganda and Tanzania) given the limitations experienced accessing workforce databases and engaging with surgical societies in these countries. Furthermore, the data collection period was taking place during the peak of the COVID‐19 pandemic and therefore it may have been difficult for those countries to engage with our requests. The project commenced in 2020 but due to the difficulties faced in collecting the data and validating this data as detailed above, data validation was not complete until 2022.

## 7 Conclusion

Increasing the surgical workforce and improving access to surgical care remains a challenge. This progress is slower than anticipated, and the non‐progression in the ratio of men to women surgeons is of note. A better understanding of the root cause of regional variation would be invaluable for workforce planning and may provide tailored solutions that could be replicated throughout the region. In addition, a more detailed understanding of surgical workload and the contribution of non‐surgeons to surgical care would give a more complete and accurate situational analysis. Retention of surgeons in Africa is high, yet overall density still needs to meet the surgical workforce requirement [15]. Future workforce planning must consider the potential growing impact of women doctors on the medical workforce [39]. Encouraging female mentorship and showcasing examples of surgical success and female leadership could drive enthusiasm for a career in surgery [28, 29]. Creating further collaborative scholarships would help increase the visibility of surgical careers in niche specialities and mitigate deterring factors for female trainees. Future aims for the COSECSA situational analysis could include creating an interactive and up‐to‐date database that is readily accessible to individual governments, and with scope to link in with international stakeholders involved in workforce planning. Finally, this study project has demonstrated the success of an international collaboration between high‐income countries (HIC) and LMICs, leading to shared reciprocal understanding and expertise. The validated database has been shared with COSECSA and will benefit member countries for future workforce planning. This model can be replicated to advance this type of research further and pursue the areas for further research identified above.

## Author Contributions

**Denise Osei‐Kuffour:** conceptualization, data curation, formal analysis, investigation, methodology, project administration, resources, software, supervision, validation, visualization, writing – original draft, writing – review and editing. **Chihena Hansini Banda:** conceptualization, data curation, formal analysis, investigation, methodology, project administration, resources, software, supervision, validation, visualization, writing – original draft, writing – review and editing. **Alice Campion:** conceptualization, data curation, formal analysis, investigation, methodology, project administration, validation, visualization, writing – original draft, writing – review and editing. **Luli Baissa Gerba:** data curation, formal analysis, investigation, methodology, project administration, validation, writing – original draft, writing – review and editing. **Precious Mutambanengwe:** data curation, formal analysis, investigation, methodology, project administration, validation, writing – original draft, writing – review and editing. **Mugisha Nkoronko:** data curation, formal analysis, investigation, methodology, project administration, validation, writing – original draft, writing – review and editing. **Michael Mwachiro:** data curation, project administration, validation, writing – original draft, writing – review and editing. **Noel Aruparayil:** conceptualization, data curation, formal analysis, investigation, methodology, project administration, supervision, validation, visualization, writing – original draft, writing – review and editing. **Alliance Niyukuri:** data curation, formal analysis, project administration, validation, visualization, writing – original draft, writing – review and editing. **Godfrey Sama Philipo:** data curation, project administration, validation, visualization, writing – original draft, writing – review and editing. **Deirdre Nally:** conceptualization, data curation, formal analysis, investigation, methodology, project administration, validation, writing – original draft, writing – review and editing. **Deirdre Mangaoang:** conceptualization, formal analysis, investigation, methodology, project administration, resources, software, supervision, validation, visualization, writing – original draft, writing – review and editing. **Eric Borgstein:** investigation, methodology, project administration, supervision, validation, visualization, writing – original draft, writing – review and editing. **Abebe Bekele:** investigation, methodology, project administration, supervision, validation, visualization, writing – original draft, writing – review and editing.

## Ethics Statement

Ethical approval provided by COSECSA and RCSI IRB.

## Conflicts of Interest

The authors declare no conflicts of interest.

## Supporting information

Table S1

Table S2

Figure S1

## Data Availability

The data that supports the findings of this study are available within the manuscript and supplementary material of this article. The full dataset has been handed over to the RCSI/COSECSA Collaborative project and is restricted.

## Appendix 1 Data Sources

- Accepted sources:
  - COSECA “Capsule” Database
  - University teaching hospitals graduate lists from each member country
  - National medical council records
  - National surgical society records
  - National Surgical, Obstetric and Anesthesia Plans (NSOAPs)
  - COSECSA and COSECSA partner event and training course participant records
  - Direct requests for information to COSECSA Council members, hospitals, hospital groups and NGOs
  - Lists of contributors to surgical journals
  - Direct contact with hospitals and surgeons
  - LinkedIn
  - Social media data collection
  - Other sources (surgical journals in the region)
  - RCSI/COSECSA/GASOC/G4 Social media campaign

## Appendix 2

See Table A2.

**TABLE A2:** Completeness of validation.

Domains	*n* = 2555	% Completeness
Count of sex	2541	99%
Count of country of practice	2555	100%
Count of country where first qualification obtained	1482	58%
Count of specialization	2427	95%
Count of affiliation to COSECSA	2465	96%
Count of hospital of primary practice	2127	83%
Count of ownership of hospital	2160	85%
Count of town/City of hospital	1800	70%
Count of validation of sources: Source type 1	2555	100%
Count of validation of sources: Source type 2	2555	100%
