# Supplementary material for: The Evolution of the Specialist Surgeon Workforce in East, Central and Southern Africa
Source: World J Surg. 2025 Mar 20;49(4):946–54. doi: 10.1002/wjs.12545 (PMC11994144; doi:10.1002/wjs.12545)
Supplement: Supplementary file 1 — Table S1 [file WJS-49-946-s002.docx]

**Supplemental Table S1: Sex differences in speciality shown with the total number of surgeons in each speciality and the proportion of women surgeons as a percentage (* : Total number of surgeons less 14 surgeons, for whom sex was not recorded)**

| **Specialty** | **Male sex** | **Female sex** | **Total** | **Proportion of women**  **surgeons (%)** |
| --- | --- | --- | --- | --- |
| General surgery | 1039 | 99 | 1138 | 9 |
| Orthopaedic surgery | 465 | 25 | 490 | 5 |
| Paediatric Surgery | 54 | 21 | 75 | 28 |
| Urology | 145 | 7 | 152 | 5 |
| Plastic Surgery | 49 | 19 | 68 | 28 |
| ENT | 176 | 44 | 220 | 20 |
| Neurosurgery | 122 | 11 | 133 | 8 |
| Cardiothoracic Surgery | 67 | 2 | 69 | 3 |
| OMFS | 62 | 7 | 69 | 10 |
| Not recorded | 119 | 8 | 127 | 6 |
| **Total:** | **2298** | **243** | **2541*** | **10** |

***Total number of surgeons less 14 surgeons, for whom sex was not recorded.
