# Supplementary material for: The Evolution of the Specialist Surgeon Workforce in East, Central and Southern Africa
Source: World J Surg. 2025 Mar 20;49(4):946–54. doi: 10.1002/wjs.12545 (PMC11994144; doi:10.1002/wjs.12545)
Supplement: Supplementary file 2 — Table S2 [file WJS-49-946-s003.docx]

| **Supplemental Table S2:** **Location of surgeon depending on population density of the region**  **** |  |  |  |  |
| --- | --- | --- | --- | --- |
