# Supplementary material for: The Evolution of the Specialist Surgeon Workforce in East, Central and Southern Africa
Source: World J Surg. 2025 Mar 20;49(4):946–54. doi: 10.1002/wjs.12545 (PMC11994144; doi:10.1002/wjs.12545)
Supplement: Supplementary file 3 — Figure S1 [file WJS-49-946-s001.docx]

**Supplemental Figures S1a – 1d: Analysis of the COSECSA Region Surgical Workforce by Sex. (a) Comparison of the proportion of Men and Women surgeons in the region in 2015 and 2022. (b) Affiliation to COSECSA. (c) Hospital of Practice. (d) Comparison of the Proportion of Women Surgeons in the region by Country in 2015 and 2022.**

**a.**

**
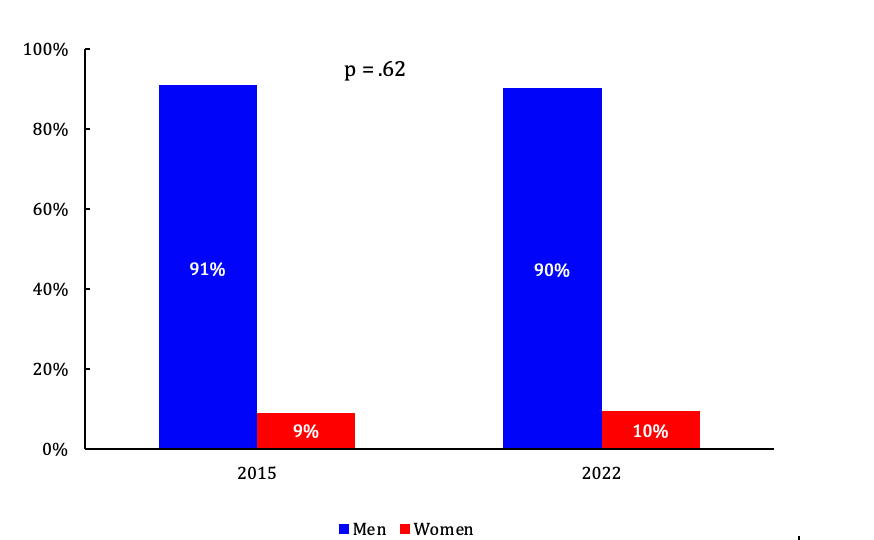
**

**b.**

**c.**

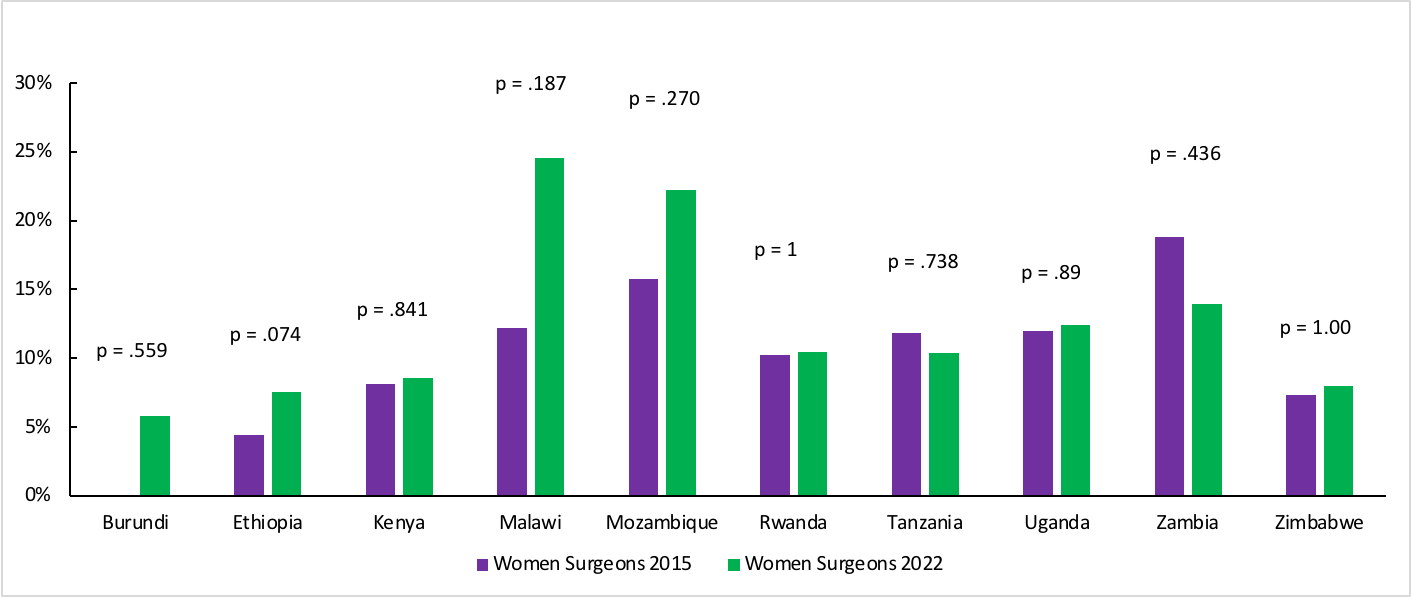
**d.**
